# Frequency of Bloodwork in Patients on Immune Checkpoint Inhibitors

**DOI:** 10.3390/curroncol33100630

**Published:** 2026-10-20

**Authors:** John R. Goffin, Sze Wah Samuel Chan, Gregory R. Pond

**Affiliations:** 1 Department of Oncology, McMaster University, 699 Concession St., Hamilton, ON L8V 5C2, Canada; 2 Department of Oncology, McMaster University, 711 Concession St., Hamilton, ON L8V 1C3, Canada

**Keywords:** immune checkpoint inhibitors, bloodwork, testing frequency, adverse events

## Abstract

**Purpose:** Immune checkpoint inhibitors (ICIs) are widely used to treat solid tumours. Prolonged use leads to increased bloodwork to monitor for adverse events (AEs). We explored the safety of decreasing bloodwork frequency while administering an ICI. **Methods:** Data was collected on patients receiving ICIs without chemotherapy for solid tumours from 2015 to 2020 at an academic cancer centre in Canada. Demographics, cancer diagnosis, ICI use, and pre-treatment bloodwork were extracted. The control group comprised all patients with all detected AEs. We modelled a decreased frequency of bloodwork by assuming that those same patients had bloodwork taken only every second or third cycle. The primary outcome was the proportion of patients with a missed bloodwork-related AE of grade 2 or higher. Descriptive statistics were used. **Results:** Among 735 patients, median age was 67, 65% were male, and most had lung cancer (39.6%) or melanoma (38.9%). Most (90.5%) received monotherapy. When neutropenia and lymphopenia were excluded, grade 2+ bloodwork-related AEs occurred in 20.9 to 27.7% of patients prior to cycle 2 of the ICI. When bloodwork is omitted every two or three cycles, respective AE miss rates were as high as 5.2 and 8.3% for thyroid abnormalities, 4.6 and 8.1% for hyperglycemia, and 1.6 and 2% for hepatitis. During the first period of bloodwork omission, overall grade 2+ AE miss rates were 10.4 to 12.4% when bloodwork was done every two cycles, and 17.9 to 23.8% when bloodwork was done every three cycles, varying by cycle duration. Miss rates were lower in later cycles. **Conclusions:** ICI bloodwork-related grade 2+ AE miss rates exceed 10% when bloodwork is omitted, suggesting that bloodwork should be obtained with each cycle of an ICI administered.

## Simple Summary

Immune checkpoint inhibitors are drugs which allow the immune system of patients to fight cancer. They have been successful in combating cancer but are often given for prolonged periods. These drugs also require monitoring with bloodwork due to the risk of side effects. Given that repeated bloodwork over months or years is unpleasant and takes laboratory time, we studied whether it is safe to do bloodwork less frequently. We looked at 735 patients who had received immune checkpoint inhibitors at a cancer centre in Canada and checked their bloodwork for side effects. We then looked at what would have happened if we had skipped every other blood test or even two out of three blood tests. We found that important side effects were missed in at least 10% of patients. We conclude that it is safest to continue doing bloodwork with each dose of an immune checkpoint inhibitor.

## 1. Introduction

Immune checkpoint inhibitors (ICIs) have been approved for a multitude of indications for malignancies over the last decade, contributing to substantial gains in survival for some patients [1]. These monoclonal antibodies target cytotoxic T-lymphocyte-associated protein 4 (CTLA-4), programmed cell death protein 1 (PD-1), and programmed cell death ligand 1 (PD-L1), proteins involved in the regulation of immune checkpoint pathways. Disruption of these checkpoints allows T cells to recognize and kill malignant cells. However, disruption of these checkpoints can also lead to immune adverse events (AEs), in which cytotoxic T cells attack normal tissues [2]. Immune AEs can occur at any time and affect any organ system, requiring careful monitoring of patients undergoing these treatments. Endocrinopathies, hepatitis, nephritis, and hematologic disturbances are possible AEs which can be detected via routine bloodwork [3]. Treatment with ICIs can continue for months or years, with adverse events occurring even months after treatment is stopped. Treatment is accompanied by equally long durations of AE monitoring.

While the cost of bloodwork is modest as compared with the current cost of immune checkpoint inhibitors, patients undergo multiple blood draws over time, along with the associated discomfort and testing time. Despite limited data, some authors have therefore suggested decreasing the frequency of bloodwork, particularly after the first three months of treatment [4,5]. It is possible that decreased bloodwork could improve the patient experience, but the question is whether doing so would pose undue risk. Using data from a large academic oncology centre, we explored the impact of bloodwork omissions on the detection of AEs.

## 2. Methods

Data was extracted from the medical records of adult oncology patients treated with their first exposure to singlet or combination immune checkpoint inhibitors at the Juravinski Cancer Centre in Hamilton, Ontario, Canada, from 1 January 2015 to 31 December 2020. Demographics, cancer diagnosis, stage, and the use of prior cytotoxic treatment were recorded. Treatment regimens included single agent atezolizumab, durvalumab, nivolumab, pembrolizumab and ipilimumab, as well as combination ipilimumab and nivolumab. Treatment and dates of treatments were captured.

Bloodwork was extracted to identify the patients with grade 2 or higher endocrinopathies, hepatitis, nephritis, and hematologic toxicities. Baseline bloodwork was derived from the most recent blood taken within 2 months of ICI start. Relevant bloodwork included a complete blood count, creatinine, glucose, alanine transaminase, aspartate transaminase, and thyroid-stimulating hormone, although individual clinicians may deviate from this selection. More targeted bloodwork such as troponin, cortisol, or rheumatologic markers was not recorded as it was typically used when pursuing specific diagnoses and was not generally collected. For cycles 2 and beyond, pretreatment bloodwork was defined as bloodwork within 5 days prior to treatment.

Bloodwork-related adverse events were based on the CTCAE version 5.0, American Society of Clinical Oncology and Cancer Care Ontario immunotherapy toxicity guidelines, and the American Diabetes Association diagnostic guideline (see Supplement Tables S1–S9) [6,7,8,9]. As symptoms were not collected, our grading was modified to be based solely on laboratory values. An adverse event was defined as worsening of AE grade as compared to baseline.

The primary outcome is the percentage of patients with a missed bloodwork-related adverse event of grade 2 or higher, excluding lymphopenia and neutropenia. At our centre, bloodwork is standardly conducted every cycle. The control group comprised all extracted patients with all detected AEs.

This retrospective counterfactual simulation modelled a decreased frequency of bloodwork (every 2 or every 3 cycles) by assuming that those same patients had no bloodwork taken every 2nd or 3rd cycle. A missed AE was thus defined as an abnormal blood test detected in the control group during a cycle in which bloodwork was (modelled as) not taken in the experimental group.

As an example, a control patient would have bloodwork prior to cycles 1, 2, and 3. That same patient modelled as an experimental patient having bloodwork only every 2 cycles would have bloodwork only prior to cycles 1 and 3. If a grade 2 or higher AE was detected during cycle 2, that would be considered a missed AE. An AE appearing only at cycle 3 would not be considered a miss.

The rate of adverse events will be evaluated assuming bloodwork assessment as follows: (1) bloodwork every cycle, (2) bloodwork every 2nd cycle, (3) bloodwork every 3rd cycle. Descriptive statistics were used to summarize the patient population, treatments received, laboratory values and adverse events experienced. Results are considered exploratory and are not to be considered definitive. This retrospective study was approved by the Hamilton Integrated Research Ethics Board, and informed consent was waived.

## 3. Results

Data was collected on 735 patients identified during the study period (Table 1). Most were male (65.7%), and the median age was 67 years. Most patients had a diagnosis of lung cancer (39.6%) or melanoma (38.9%), with a minority having genitourinary (12.0%) or head and neck (9.5%) cancers. Treatment was of curative intent in only 52 patients (7.1%), all of whom were treated for melanoma. For monotherapy, cycle lengths were every two weeks in 25.7% of patients, every three weeks in 45.4%, every four weeks in 19.1%, and every six weeks in 0.3%. Combination therapy was given every three weeks and to 9.5% of patients. Among all patients, a median of five cycles was received (range 1–83) and the median duration of treatment was 3.72 months (range 0.03–77.8 months).

Table 2 shows the grade 2+ AEs as detected by bloodwork at any time up to the cycle of treatment according to the ICI administration schedule. When neutropenia and lymphopenia are excluded (as low values are rarely attributed to immunotherapy as adverse events), AE rates appear substantially lower, and subsequent analyses in this paper exclude these cytopenias to minimize noise. Without those, any grade 2+ bloodwork-based AE occurs in 20.9 to 27.7% prior to the second cycle, with a comparable range observed in later cycles.

When bloodwork is dropped every two or three cycles, AEs arising during the excluded cycles will be missed. Table 3A,B show the miss rates for individual toxicities. Endocrinopathies are the most common toxicities. Thyroid abnormalities would be missed in 0.4–5.2% of such cases, and hyperglycemia in 1.2–4.6%. Also at the pre-cycle 2 blood test, grade 2+ hepatitis would be missed in 0–1.6%.

If bloodwork is done only every three cycles (Table 3B), numerically higher miss rates are seen through cycle 4. For example, thyroid abnormalities are missed 1.2–8.3% of the time, hyperglycemia 2.2–8.1%, and hepatitis 0–2%.

**Figure 1:**
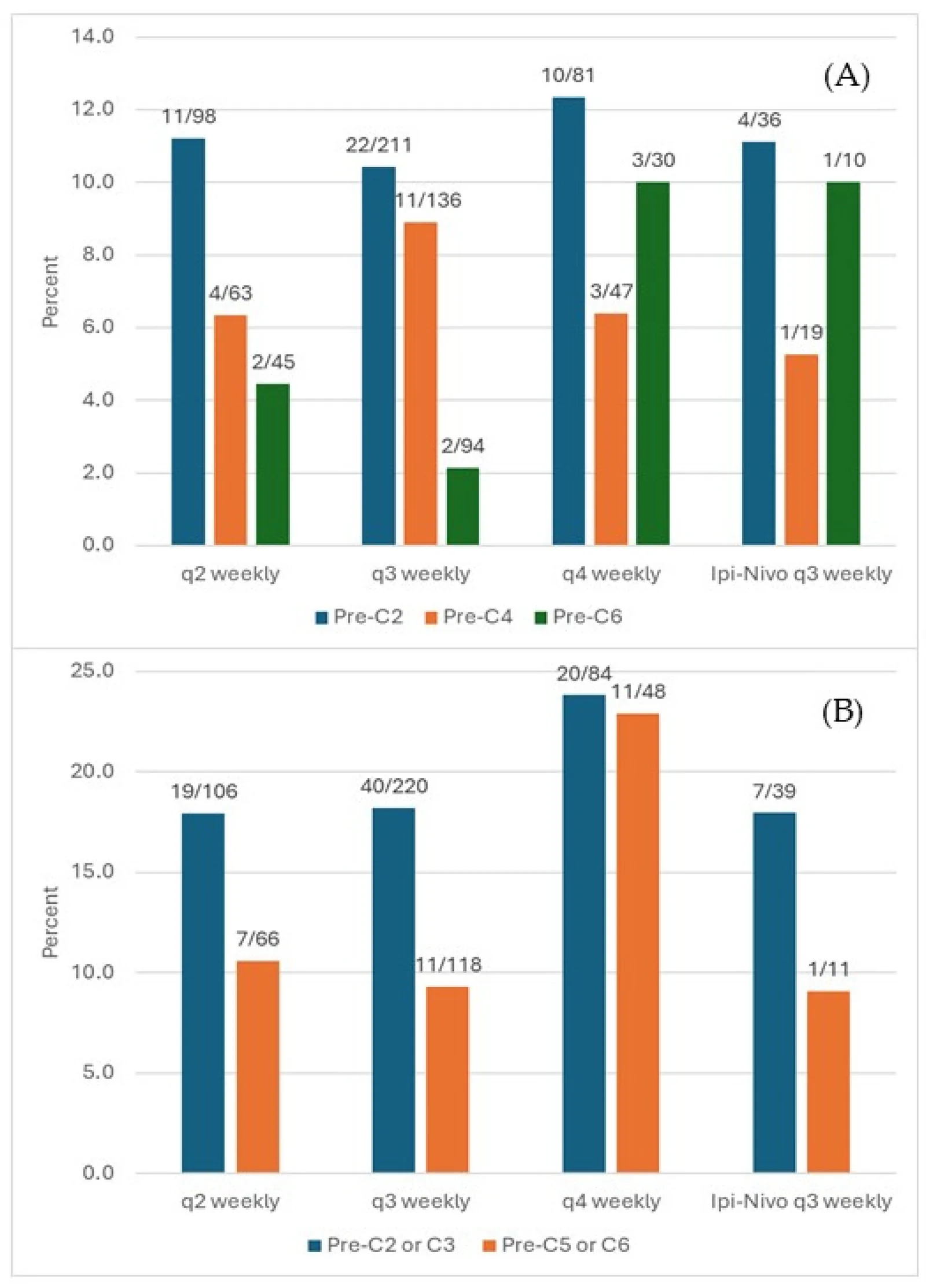
(**A**) Frequency of missed grade 2+ toxicity when bloodwork is done only every 2 cycles. (**B**) Frequency of missed grade 2+ toxicity when bloodwork is done only every 3 cycles. C2 = cycle 2; C3 = cycle 3; C5 = cycle 5; C6 = cycle 6; q2 = every 2; q3 = every 3; q4 = every 4. Note: Values exclude neutropenia and lymphopenia.

Data was sufficient to assess bloodwork omissions up to cycle 6. Rates of missed AEs are shown in Figure 1A,B, with neutropenia and lymphopenia excluded. For patients having bloodwork every second cycle, overall grade 2+ AE miss rates for bloodwork omitted at cycle 2 are 10.4 to 12.4%. If bloodwork is performed only every third cycle, the miss rate for omitting blood at cycles 2 and 3 is 17.9 to 23.8%. In every case, miss rates for bloodwork omitted at later cycles (e.g., bloodwork skipped during cycles 4, 6, or 5 + 6) are numerically lower than they are for the initial missed bloodwork. Notably, individuals with toxicities detected in earlier cycles are excluded from these later assessments as such individuals would be expected to be on heightened surveillance with no omitted bloodwork.

AE miss rates are not consistently higher for longer cycle durations.

## 4. Discussion

Immune checkpoint inhibitor use is increasing, with recent estimates in the USA suggesting that more than half of patients with incurable solid cancers are eligible for ICIs [1,10]. Expanded use and the longer survival associated with these drugs are associated with longer durations of drug administration and an increased use of oncology resources [11]. A corollary of this is an increase in time spent on treatment and testing undergone by patients, an imposition referred to as time toxicity [12]. This raises the question as to whether we can streamline therapy. One such option may be to decrease the bloodwork required of patients, if this were safe.

In this study, we assessed bloodwork-related grade 2+ AE miss rates among patients receiving ICIs when bloodwork was done only every second or third cycle. Excluding lymphopenia and neutropenia, we found miss rates above 10% for bloodwork done only every two cycles and nearer 20% for bloodwork done only every three cycles. With relatively small numbers, higher miss rates were not evident in patients receiving combination immunotherapy.

We chose grade 2+ AEs as the management of a grade 1 AE is observation in most cases while clinicians may wish to monitor more carefully if a grade 2+ AE is found [8]. Some low-grade AEs, such as thyroid dysfunction, may be safely monitored for a time without serious symptoms. Similar to white cell cytopenias, many instances of anemia will be unrelated to immunotherapy and recognition may be safely delayed. Arguably, a delay may be less important in some cases, but we were wary of excessive exclusions generating greater risk.

There is an absence of literature on acceptable rates of missed AEs. Although International Council for Harmonisation guidelines do not explicitly provide a minimum rate of AEs required for reporting, it is doubtful that the international community would consider a 10% miss rate acceptable in a clinical trial [13]. A 20% miss rate would therefore appear egregious. Nevertheless, an acceptable miss rate has not been defined.

There is a paucity of data assessing reduced bloodwork frequency. A case series describes 16 patients receiving an ICI every two weeks with variably omitted bloodwork. In that study, 43.7% of patients had bloodwork abnormalities detected by bloodwork after a time of bloodwork omission, but none was grade 3 or higher [4]. This rate appears higher than that of our study, but small numbers create uncertainty. One abstract suggested that thyroid tests may be less helpful after four cycles of treatment [14]. Conversely, Velez et al. examined patients within a California health system and found 11.6% of tested patients had thyroid dysfunction after completion of all immunotherapy, suggesting longer-term vigilance may be required [15].

Based on the above data, it seems likely that changes in drug administration are already leading to some delays in detection of bloodwork-related AEs. Recognizing the target saturation and long half-lives of ICI antibodies, longer treatment intervals have been approved. For example, while initially given every three weeks, pembrolizumab monotherapy can now be provided every six weeks at a higher dose [16]. Given that treatment frequencies have been conveniently extended to four or six weeks, it is unlikely clinicians or patients would wish to add mid-cycle bloodwork.

Both ASCO and ESMO generally recommend bloodwork be done each cycle rather than at a scheduled time interval [3,8]. Pooled trial data suggest that the time of onset of immune-related AEs is a median of 2.2 to 14.8 weeks [17], leading some to suggest that testing frequency could decrease after the first three months [5]. While our study suggests that miss rates may fall with later cycle bloodwork omissions, there was still a wide range of miss rates by cycle 5/6 (2.1 to 22.9%). Our study had insufficient data to evaluate miss rates after cycle 6, which would date to 10 to 20 weeks from the first dose depending on cycle duration.

This retrospective study has several limitations. Clinical bloodwork protocols were largely consistent, but database omissions could lead to an understatement (or, less likely, an overstatement) of miss rates. Variations in bloodwork monitoring based on prior medical conditions were not captured. The median duration of treatment was 3.72 months, hampering our ability to assess later AE rates. In this study, bloodwork was assessed without knowledge of clinical symptoms, symptoms which could lead to earlier diagnosis of AEs (requiring less reliance on bloodwork). Similarly, patients may have stopped immunotherapy due to an AE and this study cannot provide information on the relationship between bloodwork frequency and treatment discontinuation. There was less common use of longer-duration cycles during the time of this study than we would expect to be in use at present. For example, only two patients received 6-week cycles of pembrolizumab. Similarly, only 9.5% of patients received combination immunotherapy, limiting our ability to comment on these regimens.

In summary, this study found that ICI bloodwork-related grade 2+ adverse event miss rates may be greater than 10% when bloodwork is taken only every two cycles and closer to 20% when bloodwork is taken only every three cycles. Although not all of these toxicities would require immediate intervention, we propose that the numbers are too high to be acceptable. While our data are insufficient to support the addition of mid-cycle bloodwork for longer (four- or six-week) cycle lengths, they suggest that bloodwork should be obtained with each cycle of an ICI provided.

## Figures and Tables

**Table 1:** Patient characteristics.

Characteristic	Statistic	Result
*N*		735
Demographics		
Sex	*N* (%) Male	483 (65.7)
Age	Mean (sd)	66.7 (11.1)
	Median (range)	67 (25, 93)
Palliative Treatment	*N* (%)	683 (92.9%)
Regimen site group	*N* (%) GU	88 (12.0)
	Head and neck	70 (9.5)
	lung	291 (39.6)
	melanoma	286 (38.9)
Prior chemotherapy	*N* (%) Yes	362 (49.3)
Pre-Immunotherapy Lab Results		
Lymphocytes × 10^9^/L (*n* = 721)	Median (range)	1.3 (0.1, 23.4)
Neutrophils × 10^9^/L (*n* = 721)	Median (range)	5.5 (1, 170.3)
ALT U/L (*n* = 714)	Median (range)	17 (6, 198)
AST U/L (*n* = 687)	Median (range)	20 (7, 162)
Bilirubin umol/L (*n* = 714)	Median (range)	8 (2, 58)
Creatinine umol/L (*n* = 721)	Median (range)	78 (35, 384)
Glucose mmol/L (*n* = 512)	Median (range)	6 (3.2, 25.8)
Hemoglobin g/L (*n* = 721)	Median (range)	127 (73, 180)
Platelets × 10^9^/L (*n* = 721)	Median (range)	254 (53, 1230)
T4 pmol/L (*n* = 105)	Median (range)	12.2 (5.2, 30.6)
TSH mIU/L (*n* = 529)	Median (range)	1.95 (0.01, 76.04)
All Treatment Information		
Regimen	*N* (%) atezolizumab q3	4 (0.5)
	durvalumab q2	15 (2.0)
	durvalumab q4	22 (3.0)
	ipilimumab q3	40 (5.4)
	nivolumab q2	174 (23.7)
	nivolumab q4	118 (16.1)
	pembrolizumab q3	290 (39.3)
	pembrolizumab q6	2 (0.3)
	nivolumab + ipilimumab q3	70 (9.5)
Cycles	Median (range)	5 (1, 83)
Duration (in months)	Median (range)	3.72 (0.03, 77.80)
Baseline Comorbidities		
Hypothyroidism	*N* (%) Yes	73 (9.9)
Hyperthyroidism	*N* (%) Yes	5 (0.7)
Diabetes type 1	*N* (%) Yes	1 (0.1)
Diabetes type 2	*N* (%) Yes	121 (16.5)
Diabetes type unknown	*N* (%) Yes	2 (0.3)
Hepatitis B	*N* (%) Yes	1 (0.1)
Hepatitis C	*N* (%) Yes	7 (1.0)
Autoimmune hepatitis	*N* (%) Yes	1 (0.1)
Chronic kidney disease	*N* (%) Yes	27 (3.7)
Hematological malignancy	*N* (%) Yes	13 (1.8)

GU = genitourinary; q2 = every 2 weeks; q3 = every 3 weeks, etc.

**Table 2:** Toxicity rate (in percent) detected by bloodwork by cycle and treatment schedule.

Treatment	Pre-Cycle 2	Pre-Cycle 3	Pre-Cycle 4	Pre-Cycle 5	Pre-Cycle 6
Grade 2+					
q2 weekly	50/136 (36.8)	34/117 (29.1)	25/93 (26.9)	20/80 (25.0)	19/71 (26.8)
q3 weekly	78/259 (30.1)	69/220 (31.4)	53/191 (27.8)	47/160 (29.4)	28/138 (20.3)
q4 weekly	48/103 (46.6)	41/91 (45.1)	33/78 (42.3)	31/69 (44.9)	25/60 (41.7)
nivolumab ipilimumab q3	17/47 (36.2)	13/41 (31.7)	7/26 (26.9)	1/11 (9.1)	4/11 (36.4)
Grade 2+ (Excluding neutropenia and lymphopenia)					
q2 weekly	35/136 (25.7)	26/117 (22.2)	17/93 (18.3)	14/80 (17.5)	14/71 (19.7)
q3 weekly	54/259 (20.9)	55/220 (25.0)	41/191 (21.5)	36/160 (22.5)	26/138 (18.8)
q4 weekly	26/103 (25.2)	28/91 (30.8)	19/78 (24.4)	23/69 (33.3)	19/60 (31.7)
nivolumab ipilimumab q3	13/47 (27.7)	11/41 (26.8)	7/26 (26.9)	1/11 (9.1)	2/11 (18.2)

Note: Denominators diminish in later cycles as individuals with toxicity in prior cycles are excluded. q2 = every 2; q3 = every 3; q4 = every 4.

**Table 3:** (**A**) Grade 2 or higher toxicity missed when bloodwork is taken only every 2 cycles. (**B**) Grade 2 or higher toxicity missed when bloodwork is taken only every 3 cycles.

(**A**)				
Characteristic	Missed at Pre-C2 Test *N* (%)	Missed at Pre-C4 Test *N* (%)		Missed at Pre-C6 Test *N* (%)
Treatment Cycle Duration = q2 weekly				
Hypothyroid, Gr 2+	2/94 (2.1)	2/64 (3.1)		2/47 (4.3)
Hyperthyroid, Gr 2+	5/97 (5.2)	1/62 (1.6)		0/45 (0)
Diabetes, Any Gr	1/83 (1.2)	1/47 (2.1)		2/42 (4.8)
Hepatitis (ALT), Gr 2+	2/129 (1.6)	0/89 (0)		0/67 (0)
Hepatitis (AST), Gr 2+	0/131 (0)	0/90 (0)		0/68 (0)
Nephritis, any grade	0/129 (0)	0/82 (0)		0/63 (0)
Anemia, Gr 2+	6/114 (5.3)	1/74 (1.4)		0/57 (0)
Thrombocytopenia, Gr 2+	0/134 (0.0)	0/88 (0)		0/71 (0)
Treatment Cycle Duration = q3 weekly				
Hypothyroid, Gr 2+	1/225 (0.4)	3/161 (1.9)		1/114 (0.9)
Hyperthyroid, Gr 2+	5/222 (2.3)	6/150 (4.0)		0/108 (0)
Diabetes, Any Gr	6/181 (3.3)	3/124 (2.4)		1/92 (1.1)
Hepatitis (ALT), Gr 2+	1/255 (0.4)	0/188 (0)		0/132 (0)
Hepatitis (AST), Gr 2+	1/255 (0.4)	0/188 (0)		0/133 (0)
Nephritis, any grade	0/251 (0)	0/182 (0)		0/131 (0)
Anemia, Gr 2+	14/238 (5.9)	4/166 (2.4)		0/121 (0.8)
Thrombocytopenia, Gr 2+	1/257 (0.4)	0/190 (0)		0/136 (0)
Treatment Cycle Duration = q4 weekly				
Hypothyroid, Gr 2+	2/79 (2.5)	3/52 (5.8)		3/39 (7.7)
Hyperthyroid, Gr 2+	3/80 (3.8)	1/54 (1.9)		0/42 (0)
Diabetes, Any Gr	3/67 (4.5)	0/42 (0)		0/33 (0)
Hepatitis (ALT), Gr 2+	1/101 (1.0)	0/77 (0)		1/58 (1.7)
Hepatitis (AST), Gr 2+	1/101 (1.0)	0/77 (0)		0/59 (0)
Nephritis, any grade	1/99 (1.0)	0/77 (0)		0/58 (0)
Anemia, Gr 2+	2/92 (2.2)	1/67 (1.5)		0/54 (0)
Thrombocytopenia, Gr 2+	0/103 (0)	0/78 (0)		0/60 (0)
Nivolumab + Ipilimumab q3 weekly				
Hypothyroid, Gr 2+	1/45 (2.2)	1/19 (5.3)		0/11 (0)
Hyperthyroid, Gr 2+	2/44 (4.6)	3/18 (16.7)		1/11 (9.1)
Diabetes, Any Gr	2/33 (6.1)	0/13 (0)		0/11 (0)
Hepatitis (ALT), Gr 2+	0/47 (0)	0/25 (0)		1/11 (9.1)
Hepatitis (AST), Gr 2+	0/46 (0)	0/25 (0)		0/11 (0)
Nephritis, any grade	0/45 (0)	0/26 (0)		0/11 (0)
Anemia, Gr 2+	2/41 (4.9)	0/20 (0)		0/10 (0)
Thrombocytopenia, Gr 2+	0/47 (0)	0/24 (0)		0/11 (0)
(**B**)				
Characteristic	Missed at Pre-C2 or Pre-C3 Test *N* (%)		Missed at Pre-C5 or Pre-C6 Test *N* (%)	
Treatment Cycle Duration = q2 weekly				
Hypothyroid, Gr 2+	6/118 (5.1)		4/78 (5.1)	
Hyperthyroid, Gr 2+	10/120 (8.3)		2/77 (2.6)	
Diabetes, Any Gr	2/91 (2.2)		3/58 (5.2)	
Hepatitis (ALT), Gr 2+	0/141 (0)		0/94 (0)	
Hepatitis (AST), Gr 2+	1/143 (0.7)		0/93 (0)	
Nephritis, any grade	0/139 (0)		0/89 (0)	
Anemia, Gr 2+	8/123 (6.5)		0/79 (0)	
Thrombocytopenia, Gr 2+	0/145 (0)		0/95 (0)	
Treatment Cycle Duration = q3 weekly				
Hypothyroid, Gr 2+	3/252 (1.2)		2/145 (1.4)	
Hyperthyroid, Gr 2+	12/249 (4.8)		5/139 (3.6)	
Diabetes, Any Gr	11/211 (5.2)		3/129 (2.3)	
Hepatitis (ALT), Gr 2+	2/268 (0.7)		2/162 (1.2)	
Hepatitis (AST), Gr 2+	1/269 (0.4)		2/163 (1.2)	
Nephritis, any grade	0/263 (0)		0/156 (0)	
Anemia, Gr 2+	24/250 (9.6)		1/144 (0.7)	
Thrombocytopenia, Gr 2+	1/270 (0.4)		0/164 (0)	
Treatment Cycle Duration = q4 weekly				
Hypothyroid, Gr 2+	7/96 (7.3)		7/58 (12.1)	
Hyperthyroid, Gr 2+	6/97 (6.2)		2/61 (3.3)	
Diabetes, Any Gr	6/78 (7.7)		5/46 (10.9)	
Hepatitis (ALT), Gr 2+	1/107 (0.9)		1/74 (1.4)	
Hepatitis (AST), Gr 2+	1/106 (0.9)		0/74 (0)	
Nephritis, any grade	1/107 (0.9)		0/72 (0)	
Anemia, Gr 2+	3/97 (3.1)		0/65 (0)	
Thrombocytopenia, Gr 2+	0/109 (0)		0/74 (0)	
Nivolumab + Ipilimumab q3 weekly				
Hypothyroid, Gr 2+	2/49 (4.1)		0/12 (0)	
Hyperthyroid, Gr 2+	3/48 (6.3)		1/12 (8.3)	
Diabetes, Any Gr	3/37 (8.1)		0/12 (0)	
Hepatitis (ALT), Gr 2+	1/50 (2.0)		1/12 (8.3)	
Hepatitis (AST), Gr 2+	0/49 (0)		0/12 (0)	
Nephritis, any grade	0/48 (0)		0/12 (0)	
Anemia, Gr 2+	2/44 (4.5)		0/11 (0)	
Thrombocytopenia, Gr 2+	0/50 (0)		0/12 (0)	

C2 = cycle 2; C4 = cycle 4; C6 = cycle 6; q2 = every 2; q3 = every 3; q4 = every 4.

## Data Availability

The dataset in this article is not readily available due to ethics board limitations on data sharing and destruction.

## Supplementary Materials

The following supporting information can be downloaded at: https://www.mdpi.com/article/10.3390/curroncol33100630/s1, Table S1: Grading of hypothyroidism; Table S2: Grading of hyperthyroidism; Table S3: Grading of diabetes mellitus; Table S4: Grading of hepatitis; Table S5: Grading of nephritis; Table S6: Grading of anemia; Table S7: Grading of lymphopenia; Table S8: Grading of neutropenia; Table S9: Grading of thrombocytopenia.
