# Supplementary material for: Frequency of Bloodwork in Patients on Immune Checkpoint Inhibitors
Source: Curr Oncol. 2026 Oct 20;33(10):630. doi: 10.3390/curroncol33100630 (PMC13703503; doi:10.3390/curroncol33100630)
Supplement: Supplementary file 1 [file curroncol-33-00630-s001.zip › curroncol-4616459-supplementary.pdf]

Frequency of bloodwork in patients on immune checkpoint inhibitors

Supplemental Material

Adverse events table are tagged with the relevant references as follows:

ADA = American Diabetes Association diagnostic guideline[6]
ASCO = ASCO guidelines[8]
CCO = Cancer Care Ontario Guidelines[7]
CTCAE = CTCAE version 5.0[9]

Table S1. Grading of hypothyroidism

Grade	Definition
1	TSH > ULN and ≤ 10 mIU/L; free T4 normal
2	TSH > 10 mIU/L; free T4 normal
3-4	TSH > 10 mIU/L; free T4 < LLN

TSH, thyroid stimulating hormone; ULN, upper limit of normal; T4, thyroxine; LLN, lower limit of normal
TSH: 0.47 – 4.68 mIU/L
Free T4: 10.8 – 28.2 pmol/L
Based on: ASCO, CCO

Table S2. Grading of hyperthyroidism

Grade	Definition
1	TSH < LLN; free T4 normal
2-4	TSH < LLN; free T4 > ULN

TSH, thyroid stimulating hormone; LLN, lower limit of normal; T4, thyroxine; ULN, upper limit of normal
TSH: 0.47 – 4.68 mIU/L
Free T4: 10.8 – 28.2 pmol/L
Based on: ASCO, CCO

Table S3. Grading of diabetes mellitus

Grade	Definition
1-4	Random glucose ≥ 11.1 mmol/L

Based on: ADA

Table S4. Grading of hepatitis

Grade	Definition
1	AST/ALT > ULN to 3 X ULN and/or total bilirubin > ULN to 1.5 X ULN
2	AST/ALT > 3 to ≤ 5 X ULN and/or total bilirubin > 1.5 to ≤ 3 X ULN
3	AST/ALT 5-20 X ULN and/or total bilirubin 3-10 X ULN

4	AST/ALT > 20 X ULN and/or total bilirubin > 10 X ULN

ULN, upper limit of normal
ALT: 0 – 34 U/L
AST: 18 – 34 U/L
Bilirubin: 21 umol/L is considered the ULN
Based on: CTCAE

Table S5. Grading of nephritis

Grade	Definition
1	Creatinine 1.5-2 X baseline
2	Creatinine 2-3 X baseline
3	Creatinine 3-6 X baseline
4	Creatinine > 6 X baseline

Based on: ASCO, CCO

Table S6. Grading of anemia

Grade	Definition
1	Hgb < LLN to 100 g/L
2	Hgb 80-99 g/L
3-4	Hgb < 80 g/L

Hgb, hemoglobin; LLN, lower limit of normal
Based on: CTCAE

Table S7. Grading of lymphopenia

Grade	Definition
1	Lymphocyte count < LLN – 0.8 x 10^9/L
2	Lymphocyte count < 0.8 – 0.5 x 10^9/L
3	Lymphocyte count < 0.5 – 0.2 x 10^9/L
4	Lymphocyte count < 0.2 x 10^9/L

LLN, lower limit of normal
Based on: CTCAE

Table S8. Grading of neutropenia

Grade	Definition
1	Neutrophil count < LLN – 1.5 x 10^9/L
2	Neutrophil count < 1.5 – 1 x 10^9/L
3	Neutrophil count < 1 – 0.5 x 10^9/L
4	Neutrophil count < 0.5 x 10^9/L

LLN, lower limit of normal
Based on: CTCAE

Table S9. Grading of thrombocytopenia

Grade	Definition
1	Platelet count < 100/μL
2	Platelet count < 75/μL
3	Platelet count < 50/μL
4	Platelet count < 25/μL

Based on: CTCAE

References

6. American Diabetes Association. Diagnosis and classification of diabetes mellitus. *Diabetes Care* **2014**, *37 Suppl 1*, S81–90, doi:10.2337/dc14-S081.
8. Schneider, B.J.; Naidoo, J.; Santomasso, B.D.; Lacchetti, C.; Adkins, S.; Anadkat, M.; Atkins, M.B.; Brassil, K.J.; Caterino, J.M.; Chau, I.; et al. Management of Immune-Related Adverse Events in Patients Treated With Immune Checkpoint Inhibitor Therapy: ASCO Guideline Update. *J Clin Oncol* **2021**, *39*, 4073–4126, doi:10.1200/JCO.21.01440.
7. Cancer Care Ontario Expert Working Group. Immune Checkpoint Inhibitor Toxicity Management Clinical Practice Guideline. Available online: https://www.cancercareontario.ca/en/guidelines-advice/types-of-cancer/52976
9. U.S. Department of Health and Human Services. Common Terminology Criteria for Adverse Events Version 5.0. Available online: https://dctd.cancer.gov/research/ctep-trials/for-sites/adverse-events/ctcae-v5-5x7.pdf
